# The effect of end-tidal carbon dioxide on optic nerve diameter in COPD patients: A cross-sectional study

**DOI:** 10.1097/MD.0000000000046570

**Published:** 2025-12-12

**Authors:** Zuhal Ozer Simsek, Kaniye Aydin, Seda Guzeldag, Merva Tuna

**Affiliations:** a Department of Chest Intensive Care Unit, Kayseri City Hospitals, Kayseri, Turkey; b Department of Internal Medicine Intensive Care, Kayseri City Hospitals, Kayseri, Turkey; c Department of Neurology, Kayseri City Hospitals, Kayseri, Turkey; d Department of Neurology Intensive Care Unit, Kayseri City Hospitals, Kayseri, Turkey.

**Keywords:** COPD, headache, hypercarbia, optic nerve diameter

## Abstract

Chronic obstructive pulmonary disease (COPD) is a heterogeneous condition that causes persistent and often progressive airway obstruction with symptoms such as dyspnea, cough, expectoration, and/or exacerbations due to abnormalities in the airways and/or alveol. It is known that hypercarbia causes headaches by increasing intracranial pressure (ICP). Ultrasonographic measurement of optic nerve sheath diameter (ONSD) noninvasively has been shown to be as successful as invasive measurements in demonstrating increased intracranial pressure. The aim of this study was to determine the effect of the decrease in end-tidal carbon dioxide pressure on the optic nerve diameter and thus get an idea about ICP and its correlation with symptoms such as headache in hypercarbic patients with severe exacerbations of COPD. Patients with COPD ICD codes who needed noninvasive mechanical ventilation (NIMV) were included in the study. Patients who were incompatible with NIMV or who were switched to invasive mechanical ventilation due to treatment failure were excluded from the study. The decrease in end-tidal carbon dioxide, the change in ONSD, in the state of consciousness, and in symptoms such as headache was evaluated. There was no statistically significant difference in optic nerve diameter between those with and without headache at baseline. This difference was not significant: 6.45 (6–6.7) mm in those with ONSD headache and 6.4 (6–6.7) mm in those without. As a result, although the ONSD was wider than expected in patients with hypercarbic COPD, no relationship was found with headache.

## 1. Introduction

Chronic obstructive pulmonary disease (COPD) is a heterogeneous condition that causes persistent and often progressive airway obstruction with symptoms such as dyspnea, cough, expectoration, and/or exacerbations due to abnormalities in the airways and/or alveoli.^[1]^ Exacerbations of COPD (ECOPD) are an important condition associated with disease progression and a poor prognosis, leading to increased use of health resources. Severe ECOPD is defined as showing new onset/worsening hypercapnia and acidosis; partial pressure of arterial carbon dioxide (PaCO2) >45 mm Hg and pH <7.35 in arterial blood gas. It is recommended to use noninvasive mechanical ventilation (NIMV) primarily in patients who require ventilatory support due to severe exacerbations.^[2]^ A decrease in partial carbon dioxide pressure and improvement in consciousness are expected conditions for early treatment success in patients undergoing noninvasive mechanical ventilation due to an exacerbation of COPD.^[3]^ It is known that hypercarbia causes headaches by increasing intracranial pressure (ICP).^[4]^ Ultrasonographic measurement of optic nerve sheath diameter (ONSD) noninvasively has been shown to be as successful as invasive measurements in demonstrating increased intracranial pressure.^[5]^ Although there are minimal differences in various studies in the literature, it has been shown that the cut point for ONDS of 5.1 mm (the ideal cut point of 5.3 mm for sensitivity, 75% specificity, and 91% specificity) is clinically useful in demonstrating ICP.^[6]^

In hypercarbic patients undergoing NIMV due to severe COPD exacerbation, a decrease in carbon dioxide pressure may lead to a decrease in ICP, leading to an improvement in symptoms, especially headaches, and in consciousness. The aim of this study was to determine the effect of the decrease in end-tidal carbon dioxide pressure on the optic nerve diameter and thus get an idea about ICP and its correlation with symptoms such as headache in hypercarbic patients with severe ECOPD. Headache was graded as low (1–3), moderate (4–6), and severe (7–10) using the numeric rating scale (NRS).^[7]^

## 2. Materials and methods

The study was conducted prospectively in the tertiary chest diseases intensive care unit between January 2022 and 2023. The Ethical Approval was taken from the local ethics committee as human subjects study with decision number of 2019/580. Patients with COPD who needed NIMV were included in the study. Patients who were incompatible with NIMV or who were switched to invasive mechanical ventilation due to treatment failure were excluded from the study. Comorbidities and conditions that may affect the optic nerve diameter such as diabetic patients at risk of retinopathy, severe hypertensive patients, acute stroke, intracranial hemorrhage, brain tumor, body mass index >40 kg/m², hypo-hypernatremia, hyperglycemia were not included in the study. End-tidal karbondioksit (EtCO_2_) measurements were made at the bedside by monitoring with a Medtronic CapnostreamTM 35 portable respiratory monitor delivered from the U.S. On admission, arterial blood gas was taken, optic nerve diameter was measured, and end-tidal carbon dioxide monitoring was performed. The same measurements were repeated at the lowest end-tidal carbon dioxide value observed within 24 hours of starting the NIMV application. As a result of the decrease in end-tidal carbon dioxide, the change in ONSD, in the state of consciousness, and in symptoms such as headache was evaluated. Headache was graded as low (1–3), moderate (4–6), and severe (7–10) using the NRS.^[7]^ Patients with cooperation were asked to rank their headaches numerically from 1 to 10.

### 2.1. Ultrasonography technique

The L12–4 MHz linear probe of the Philips Clear-Vue Ultrasound instrument was used for evaluation, and the same radiologist provided blinded ONSD measures for each eyeball, 3 mm below the papillae, in line with the literature. The participants were supine with their heads raised by roughly 30 degrees. The superior-outer temporal area of the upper eyelid was the location of the probe. The assessments were conducted 3 mm behind the retina in both eyes using ultrasound gel on the surface of each eyelid. The measurements were taken in the axial and sagittal planes of the largest diameter visible. Three measurements were made for the right eye and 3 measurements were made for the left eye from each subject.

### 2.2. Statistical analysis

Continuous data were summarized as median and quartiles (Q1–Q3), and categorical variables were expressed as numbers and percent ages. The normality of the distribution for continuous variables was confirmed using the Shapiro–Wilk test. The Wilcoxon test was used to compare the change blood gase analysis, OND and GCS (Glasgow coma scale) between baseline and after noninvasive mechanical ventilation. All of the analyses were carried out using IBM SPSS 20.0 (Armonk, NY: IBM Corp.). A *P*-value of <.05 was considered to show a statistically significant result.

## 3. Results

This study included 30 patients, 6 women and twenty-four men. The average age was 67.5 years. The APACHE II score was 16 (12.75–20). The majority of the patients were hospitalized in the emergency department (93.3%) Hypertension was the most prevalent comorbidity (Table 1).

**Table 1:** Demographic characteristics.

	n = 30
Age (years), median (Q1–Q3)	67.5 (55–77.25)
Gender, n (%)	
Female	6 (20)
Male	24 (80)
BMI (kg/m^2^), median (Q1–Q3)	26.34 (21.81–31.06)
APACHE II score	16 (12.75–20)
Admission to ICU, n (%)	
From emergency department	28 (93.3)
From inpatients service	2 (6.7)
Comorbidities, n (%)	
Hypertension	4 (13.3)
Heart failure	3 (10)
Cerebrovascular event	2 (6.7)

APACHE II score = acute physiologic and chronic health evaluation, BMI = body mass index, ICU = intensive care unit.

The patients’ initial pH levels changed from acidosis (mean 7.28) to the normal range (mean 7.41) (*P* <.001). The average partial pressure of carbon dioxide decreased from 81.6 to 63 mm Hg (*P* <.001). End-tidal CO2 measurement regression decreased from 80 to 60 mm Hg (*P* <.001) After the decrease in end-tidal carbon dioxide, the change in ONSD decreased from 6.4 mm (6–6.7) to 5.85 mm (5.28–6.15); this difference was statistically significant (*P* <.001). Correlation analysis showed no significant linear relationship between the magnitude of EtCO_2_ reduction and ONSD change. The GCS was initially (Q1–Q3) 14 (13–14) and showed a statistically significant complete recovery in all patients with NIMV application (*P* <.001). (Table 2). NIV was applied in line with guideline recommendations that borderline impaired consciousness due to reversible hypercapnia is not an absolute contraindication.^[3]^ All such patients showed recovery of GCS after EtCO_2_ reduction.

**Table 2:** Initial and subsequent measurements and values.

	Initial	Last	*P*-value
pH, (Q1–Q3)	7.28 (7.25–7.33)	7.41 (7.37–7.47)	**<.001**
PCO_2_ (mm Hg), (Q1–Q3)	81.6 (76.7–87.1)	63 (55–66)	**<.001**
PO_2_ (mm Hg), (Q1–Q3)	45.7 (36.7–58.0)	60 (44.5–76.3)	**.048**
SpO_2_ (%), (Q1–Q3)	80.5 (62.9–88.8)	92 (85–95.8)	**.002**
HCO_3_, (Q1–Q3)	33 (29.2–38)	35.8 (31–38.5)	**.052**
Lactate (mmol/L), (Q1–Q3)	0.9 (0.68–1.43)	1.2 (0.9–1.6)	**.169**
BUN (mg/dL), (Q1–Q3)	19.5 (15.5–29.8)	–	–
Creatinine (mg/dL), (Q1–Q3)	0.78 (0.5–0.97)	–	–
Sodium (mmol/L), (Q1–Q3)	139.5 (137.3–142)	–	–
Potassium (mmol/L), (Q1–Q3)	4.9 (4.4–5.3)	–	–
EtCO_2,_ (Q1–Q3)	80 (74.8–84.3)	60 (53.3–64)	**<.001**
OND, (Q1–Q3)	6.4 (6–6.7)	5.85 (5.28–6.15)	**.001**
GCS, (Q1–Q3)	14 (13–14)	15 (15–15)	**<.001**
Headache, n(%)	10 (33.3)	-	-

Bold values indicate *P* < .05 was considered statistically significant. GCS = Glasgow coma scale, HCO_3_ = bicarbonate, OND = optic nerve diameter, PCO_2_ = partial pressure of carbon dioxide, PO_2_ = partial pressure of, oxygen, SpO_2_ = peripheral arterial oxygen saturation.

At baseline, 29 patients (96.7%) had ONDs >5.1 mm, with only 10 (34.5%) experiencing headaches. There were no headaches in patients whose GCS was not full due to hypercarbia but improved after NIMV treatment.

At baseline, ONSD was measured as 6.45 (6–6.7) mm in those with headaches and 6.4 (6.0–6.7) mm in those without headaches. This difference was not statistically significant.

## 4. Discussion

This study shows that ONSD is elevated in hypercarbic COPD exacerbations and significantly decreases after EtCO_2_ reduction. However, the absence of correlation with headache suggests that headache is not a reliable surrogate for ICP in this population. Instead, reversible hypercapnia-induced cerebral hemodynamic changes may underlie consciousness improvement, consistent with previous findings.

Severe COPD exacerbations are characterized by PaCO2 levels above 60 mm Hg, pH levels below 7.25, excessive use of accessory muscles, and abnormal mentation.^[1]^ Noninvasive mechanical ventilation is one of the first treatment options for severe COPD exacerbations that do not respond to optimal medical therapy.^[8]^ Severe COPD exacerbations accompanied by hypercapnic respiratory failure have been shown to be associated with more severe disease with higher mortality.^[9]^

Central and peripheral chemoreceptors that regulate breathing in the central nervous system are sensitive to variations in blood PO2, PCO2, and pH.^[10]^ In the presence of hypoxia or hypercarbia, cerebral blood flow increases as a result of dilation of cerebral blood vessels.^[11]^ As a result, in respiratory failure, especially in COPD, many neurological symptoms such as headaches, abnormal movements, and mental status changes can be observed as a result of changes in cerebral blood flow.^[12]^ The hypothesis for this study was that increased intracranial pressure caused by increasing carbon dioxide would result in headaches in hypercapnic COPD patients.

One study investigated the relationship between high altitude hypoxemia and elevated ICP, and thus the neurological symptoms of acute mountain sickness.^[13]^ Direct ICP measurements were compared to headache scores. There was no significant result identified. According to our findings, ONSD was higher than the cutoff value established in the literature^[6]^ in almost all COPD patients who received NIMV. However, only 34.5% of these patients with high ONS experienced headaches. ONSD showed no significant difference between those with and without headaches. The diameter of the ONSD decreased significantly when the patient’s carbon dioxide levels decreased. According to these findings, there is no significant link between increased ONSD and headaches in COPD patients. Although this considerable decline in ONSD was not found to be associated with headache, it may have an impact on GKS and certain cognitive functions. Studies on bigger patient populations may be required to confirm this. In another study, it has been shown that the parameters mainly responsible for neurological deterioration in acute respiratory failure are pCO2 and pH rather than pO2.^[14]^ Lowering pCO2 has been shown to improve neurological deterioration, whereas increasing pO2 has no such benefit. In the current investigation, when the basic follow-up parameter, pCO2, decreased, all patients earned complete GCS.

To our knowledge, this is one of the few studies looking into the increase in intracranial pressure caused by increased carbon dioxide in COPD attacks that require noninvasive mechanical ventilation assistance. Although it has not been demonstrated that headaches are connected with higher ONSD and, as a result, increased ICP, this study is one of the few that evaluate transorbital USG and ICP and their correlation with symptoms of carbon dioxide retention necessitating NIMV for COPD.

Limitations of this study were that ICP was not directly measured (ONSD was used as a validated noninvasive surrogate), the sample size was small, and there was no long-term neurological follow-up.

## 5. Conclusion

In conclusion, ONSD is significantly elevated in hypercarbic COPD exacerbations and decreases after EtCO₂ reduction, reflecting reversible intracranial pressure changes. However, no direct correlation was found with headache. Further studies with larger cohorts are warranted.

## Author contributions

**Conceptualization:** Zuhal Ozer Simsek, Kaniye Aydin, Seda Guzeldag.

**Data curation:** Zuhal Ozer Simsek.

**Formal analysis:** Zuhal Ozer Simsek, Kaniye Aydin.

**Investigation:** Zuhal Ozer Simsek.

**Methodology:** Zuhal Ozer Simsek, Kaniye Aydin, Seda Guzeldag, Merva Tuna.

**Resources:** Zuhal Ozer Simsek.

**Software:** Zuhal Ozer Simsek.

**Validation:** Zuhal Ozer Simsek.

**Visualization:** Zuhal Ozer Simsek.

**Writing – original draft:** Zuhal Ozer Simsek.

**Writing – review & editing:** Zuhal Ozer Simsek.

## References

[1] Agustí A Celli BR Criner GJ. Global initiative for chronic obstructive lung disease 2023 report: GOLD executive summary. Am J Respir Crit Care Med. 2023;207:819–37. 36856433 10.1164/rccm.202301-0106PP PMC10111975

[2] Celli BR Fabbri LM Aaron SD. An updated definition and severity classification of COPD exacerbations: the Rome proposal. Am J Respir Crit Care Med. 2021;204:1251–8. 34570991 10.1164/rccm.202108-1819PP

[3] British Thoracic Society. 2019: BTS National Audit Report: Adult NIV Audit 2019. 1 February - 31 March. 2019. https://www.brit-thoracic.org.uk/news/2020/bts-adult-non-invasive-ventilation-audit-2019/ Accessed: November 8 2020.

[4] Canakci Y Koksal O Durak VA. The value of bedside ocular ultrasound assessment of optic nerve sheath diameter in the detection of increased intracranial pressure in patients presenting to the emergency room with headache. Niger J Clin Pract. 2018;21:778–82. 29888727 10.4103/njcp.njcp_119_17

[5] Lau T Ahn JS Manji R Kim DJ. A narrative review of point of care ultrasound assessment of the optic nerve in emergency medicine. Life (Basel). 2023;13:531. 36836888 10.3390/life13020531 PMC9962087

[6] Kim DY Kim SY Hong DY. Comparison of ultrasonography and computed tomography for measuring optic nerve sheath diameter for the detection of elevated intracranial pressure. Clin Neurol Neurosurg. 2021;204:106609. 33813371 10.1016/j.clineuro.2021.106609

[7] Hong CK Joo JY Shim YS. The course of headache in patients with middle-to-high headache due to low traumatic brain injury: a retrospective cross-sectional study. J Headache Pain. 2017;18:48. 28429236 10.1186/s10194-017-0755-9 PMC5398969

[8] MacIntyre NR. Acute hypercapnic respiratory failure in COPD. Respir Care. 2023;68:973–82. 37353327 10.4187/respcare.10560 PMC10289623

[9] Chen L Chen L Zheng H Wu S Wang S. Emergency admission parameters for predicting in-hospital mortality in patients with acute exacerbations of chronic obstructive pulmonary disease with hypercapnic respiratory failure. BMC Pulm Med. 2021;21:258. 34362328 10.1186/s12890-021-01624-1 PMC8349105

[10] Eldridge F. Central nervous system and chemoreceptor factors in control of breathing. Chest. 1978;73:256–8. 23257 10.1378/chest.73.2_supplement.256

[11] Posner JB. Plum and Posner’s Diagnosis of Stupor and Coma. Oxford University Press; 2007.

[12] Dreibelbis JE Józefowicz RF. Neurologic complications of respiratory disease. Neurol Clin. 2010;28:37–43. 19932374 10.1016/j.ncl.2009.09.005

[13] Wilson MH Milledge J. Direct measurement of intracranial pressure at high altitude and correlation of ventricular size with acute mountain sickness: brian Cummins’ results from the 1985 Kishtwar expedition. Neurosurgery. 2008;63:970–4; discussion 974. 19005388 10.1227/01.NEU.0000327885.15132.CA

[14] Kilburn KH. Neurologic manifestations of respiratory failure. Arch Intern Med. 1965;116:409–15. 14325915 10.1001/archinte.1965.03870030089015

